# Nonlinear mixed models to describe the growth of Columbia sheep in Mexico

**DOI:** 10.1093/tas/txag100

**Published:** 2026-07-17

**Authors:** Ricardo Martínez-Rocha, Rodolfo Ramírez-Valverde, Rafael Núñez-Domínguez, Joel Domínguez-Viveros, Víctor Díaz Sanchez, Jonathan Valerio-Hernández, Katherine Scheflen, Jorge Hidalgo

**Affiliations:** Posgrado en Producción Animal, Universidad Autónoma Chapingo, Texcoco, 56230, México; Posgrado en Producción Animal, Universidad Autónoma Chapingo, Texcoco, 56230, México; Posgrado en Producción Animal, Universidad Autónoma Chapingo, Texcoco, 56230, México; Facultad de Zootecnia y Ecología, Universidad Autónoma de Chihuahua, Chihuahua, 31453, México; Facultad de Estudios Superiores Cuautitlán, Universidad Nacional Autónoma de MéxicoCuautitlán Izcalli, Mexico, 54714, México; Facultad de Medicina y Veterinaria y Zootecnia, Universidad Nacional Autónoma de México, Ciudad Universitaria, Ciudad de México, 04510, Mexico; Department of Animal and Dairy Science, University of Georgia, Athens, GA 30602, United States; Department of Animal and Dairy Science, University of Georgia, Athens, GA 30602, United States

**Keywords:** growth functions, variance components, growth curve modeling

## Abstract

Our objective in this study was to identify the nonlinear mixed model that best describes the growth curve of Columbia sheep within the central region of Mexico. The study was conducted using a total of 1886 live body weight records (birth to 450 days of age) from 174 Columbia Sheep (90 males and 84 females). Only animals with at least four weight records were considered in the study. The sheep were fed twice daily with a ration of corn silage and oat hay. Animals were weaned at an average age of 75 days. Growth curves were fitted using four nonlinear mixed models: Brody, Logistic, Von Bertalanffy, and Gompertz. In all models, the asymptotic body weight (A), integration constant (b), and maturity rate (k) were estimated, with A and k modeled as both fixed and random effects. Sex was included as a fixed effect affecting only the A and k parameters. A model comparison was performed using the Akaike Information Criterion (AIC), which states that lower AIC indicates better fit. Among the evaluated models, the Von Bertalanffy model provided the best fit for the data. Estimated parameters for females were A = 40.26 kg, b = 0.516, and k = 0.011 day^−1^, while the sex effects for males were + 9.55 kg for A and −0.001 for k. These estimates indicate that males reach higher mature body weights while maturing slightly more slowly than females. Including random effects for A and k improved model accuracy by reducing residual variance. These results provide useful information on growth characterization may help define age or weight targets for further evaluation of slaughter strategies in Columbia sheep.

## Teaser

The Von Bertalanffy model best fitted growth trends in Columbia sheep, revealing that males achieve higher mature weights but mature more slowly than females. Farmers can improve feeding strategies and optimize slaughter age in sheep production systems.

## Introduction

A growth curve describes how a biological variable, such as body weight, changes over time, capturing the dynamic process of growth from early development to maturity. The use of nonlinear models allows this pattern to be represented more realistically. Growth typically accelerates, reaches an inflection point, and then slows as it approaches an asymptotic maximum. This trend facilitates the interpretation and comparison of growth patterns across individuals, breeds, or management conditions.

Additionally, growth models are valuable tools for estimating daily nutritional requirements, evaluating the impact of environmental factors on weight gain, establishing genetic selection strategies, and predicting optimal slaughter age (Teleken et al. 2017). Moreover, they help to determine the optimal marketing time, thereby improving the economic efficiency of production systems. Growth curves allow for the estimation of key biological parameters, such as mature weight (*A*), growth rate (*k*), and the inflection point (*b*), which are crucial for both research and management purposes (Makgopa et al. 2023).

Identifying the best-fitting model for describing growth curves is essential for providing objective and precise information on the growth patterns of sheep and other domestic animals. Breeders can use this information to validate decisions related to production, management, and genetic selection (Domínguez-Viveros et al. 2019).

Although nonlinear growth models have been evaluated in several sheep breeds, information for Columbia sheep raised under commercial production systems in Mexico is scarce. Differences in genotype, environmental conditions, and management practices may affect growth dynamics and the biological interpretation of model parameters. Therefore, evaluating the performance of nonlinear mixed-effects models in this population provides useful information for describing growth patterns and deriving biologically meaningful growth indicators that may support management decisions.

Therefore, our objective in this study was to identify the best-fitting growth curve by comparing four nonlinear models for female and male Columbia sheep from the central region of Mexico and to derive biologically meaningful growth indicators that may support management decisions for Columbia sheep under Mexican production conditions.

## Materials and methods

A total of 174 Columbia sheep (90 males and 84 females) were included in the analysis. The flock consisted of purebred Columbia sheep maintained under a commercial production system. Live body weight was recorded repeatedly throughout the animals’ lives, yielding a total of 1,886 measurements (1117 for males and 769 for females). The average of records per animal was 10.84 (Fig. S1). Observations from birth to 450 days of age were considered, including only sheep with more than three weight records (Fig. S2).

The data were recorded from a nucleus flock of Columbia sheep in the State of Mexico. Animals belonged to the experimental flock of National Autonomous University of Mexico. Records were collected between 2023 and 2024. Animals were fed twice daily with corn silage and oat hay. Weaning occurred at an average age of 75 days. Male and female lambs were managed together until weaning and separately thereafter. Anthelmintic treatment was administered only to animals with high FAMACHA© scores as part of a targeted selective treatment strategy.

Growth curve analyses were performed using nonlinear mixed-effects models to describe body weight changes over time. The nonlinear growth functions evaluated were Brody, Logistic, Von Bertalanffy, and Gompertz. In general form, the models were expressed as:


$$\mathrm{Brody}:\;y_{ij}=f\;(t_{ij};\phi_{i})+\varepsilon_{ij}=A_{i}(1-b\;e^{-k_{i}t_{ij}})+\varepsilon_{ij}$$

$${\mathrm{Logistic}:\;y}_{ij}=f\;(t_{ij};\phi_{i})+\varepsilon_{ij}=A_{i}/(1+b\;e^{-k_{i}t_{ij}})+\varepsilon_{ij}$$

$$\mathrm{Von}\;\mathrm{Bertalanffy}:y_{ij}=f\;(t_{ij};\phi_{i})+\varepsilon_{ij}=A_{i}{\left(1-b\;e^{-k_{i}t_{ij}}\right)}^{3}+\varepsilon_{ij}$$

$$\mathrm{Gompertz}:\;y_{ij}=f\;(t_{ij};\phi_{i})+\varepsilon_{ij}=A_{i}e^{-b\;e^{-k_{i}t_{ij}}}+\varepsilon_{ij},$$

where $y_{ij}$ is the body weight of animal imeasured at the j-th observation, $t_{ij}$ is the corresponding age (days), and $\varepsilon_{ij}$ is the residual error.

The model parameters were defined as follows: $\phi_{i}=\phi_{p}+\eta_{i}$, where $\phi_{p}={\left(A_{0}+A_{\mathrm{sex}},\;b,\;k_{0}+k_{\mathrm{sex}}\right)}^{T}$ is the vector of population-level fixed effects and $\eta_{i}={\left(\eta_{A_{i}},0,\eta_{k_{i}}\right)}^{T}$ is the vector of animal-specific random effects, assuming:


$$\eta_{i}∼N(0,\Omega )$$

$$e_{i}\;∼\;N\;(0,\;\sigma^{2})$$

Where


$$\Omega =(\begin{array}{cc} \sigma_{A}^{2} & \sigma_{Ak}\\ \sigma_{Ak} & \sigma_{k}^{2} \end{array})$$

The fixed-effect structure was established through a sequential model selection procedure. The sex effect on parameter *b* was not statistically significant (*P* > 0.05) in any of the four nonlinear models; therefore, the asymptotic body weight (A) and the maturity rate (k) were modeled as sex-dependent fixed effects, and the integration constant (b) was assumed to be common across sexes. Accordingly, fixed effects were specified as: $A=A_{0}+A_{\mathrm{sex}}$ and $k=k_{0}+k_{\mathrm{sex}}$, where $A_{\mathrm{sex}}$ and $k_{\mathrm{sex}}$ represent the fixed effect of sex on the corresponding growth parameters.

Parameters and(co)variance components were estimated via maximum likelihood using the nlminb optimizer within the *nlme* package (Pinheiro et al. 2016) in R. The convergence tolerance was set to 10^−6^ with a maximum of 200 iterations. First-order continuous autoregressive and power variance structures were tested but excluded due to severe model overparameterization, as the convergence criterion was not reached. Consequently, residual independence and homoscedasticity were assumed. To determine the most appropriate growth model, goodness-of-fit criteria was evaluated using the Akaike Information Criterion (AIC) and the Root Mean Square Error (RMSE).

Growth indicators were calculated derived from the estimated parameters of the selected best-fitting model: age (AIP) and body weight (WIP) at the inflection point (except for the Brody model), age at 50% of maturity (A50M), percentage of maturity at 5 months old (PM150d) and age to reach 40 kg (A40).

## Results

Estimates of model parameters and variance components for the four nonlinear mixed growth models are presented in Table 1.

**Table 1:** Comparison of nonlinear mixed growth models and parameter estimates for body weight data.

Parameter	Von Bertalanffy	Gompertz	Brody	Logistic
A	40.26 (36.643, 43.649)	38.55 (35.106, 41.888)	44.62 (39.578, 49.951)	36.15 (33.325,38.985)
A (sex)	9.55 (5.031, 14.939)*	4.58 (0.005, 9.283)*	5.53 (−0.492, 13.657)	3.60 (−0.253, 7.464)
b	0.5162 (0.512, 0.530)	2.031 (2.008, 2.104)	0.915 (0.915, 0.928)	5.18 (4.945, 5.426)
k	0.011 (0.009,0.012)	0.013 (0.012,0.014)	0.006 (0.005, 0.007)	0.021 (0.020, 0.023)
k (sex)	−0.001 (−0.003, −0.0002)*	9e−5 (−0.001, 0.001)	7e-5 (−0.001, 0.001)	5e−4 (−0.001, 0.002)
$\sigma_{A}^{2}$	147.211	150.246	247.023	109.941
$\sigma_{k}^{2}$	1e−5	1e−5	7e−6	1.9e−5
$\sigma_{\varepsilon }^{2}$	5.380	5.551	5.672	6.135
**Cor (A, k)**	−0.622	−0.701	−0.787	−0.643
**AIC**	7637.359	7654.812	7683.607	7775.704
**ΔAIC**	–	17.453	46.248	138.345
**RMSE**	2.154	2.193	2.201	2.309

A = asymptotic body weight; b = integration constant; k = maturity rate; σ^2^ = variance component; Cor(A, k) = correlation between random effects; RMSE = Root Mean Square Error.

* Indicates statistical significance at *P* < 0.05.

**Figure 1:**
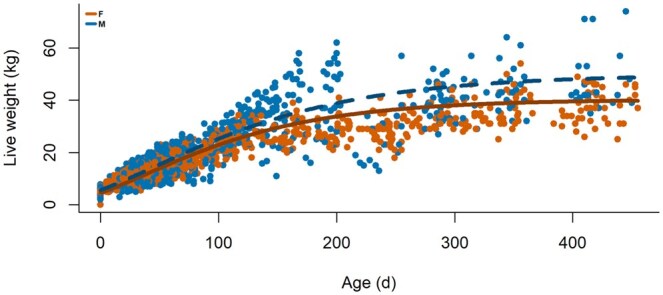
Fitted nonlinear mixed effects Von Bertalanffy growth curves for Columbia sheep by sex.

Numerical convergence was successfully achieved for all four non-linear growth models evaluated. Based on the Akaike Information Criterion (AIC), the Von Bertalanffy model exhibited the lowest value, indicating stronger empirical support than the Gompertz, Logistic, and Brody models. This superior performance was further validated by residual diagnostics and prediction accuracy metrics, as the Von Bertalanffy model yielded the lowest Root Mean Square Error (RMSE) Consequently, the Von Bertalanffy model was selected as the most appropriate framework to describe the growth curve. Therefore, the Von Bertalanffy model was selected to describe the body weight changes of Columbia sheep over time (Fig. 1).

The estimated asymptotic body weight (A) for females under the Von Bertalanffy model was 40.26 kg. A significant sex effect on asymptotic weight was detected, with males exceeding females by 9.55 kg (*P* < 0.05). The estimated maturity rate (k) for females was 0.011 day^−1^, and a small yet significant sex effect was observed, with males presenting a lower k value (−0.001; *P* < 0.05).

The variance components associated with the random effects of A and k indicated substantial individual variability in growth parameters. A negative correlation between the random effects of A and k was observed across all evaluated models, with values ranging from −0.622 to −0.787.

Growth curves fitted using the Von Bertalanffy model are shown in Fig. 1. Derived growth indicators calculated from the estimated parameters of the Von Bertalanffy are presented in Table 2. Females exhibited lower age and body weight at the inflection point (AIP and WIP) than the males. According to the Von Bertalanffy model, male Columbia lambs reached approximately 40 kg in body weight at 212 days of age under the studied production system.

**Table 2:** Growth indicators derived from the estimated parameters of the Von Beratlanffy model for female and male Columbia sheep.

Indicators	Equation	Value
**Females**		
** AIP (d)**	ln(3b)/k	39.61
** WIP (kg)**	8A/27	11.93
** A50M (d)**	$-\ln((0.5^{\frac{1}{3}}-1)/-b)/k$	83.06
** PM150d (%)**	${\left(1-be^{\left(-k(150)\right)}\right)}^{3}$	73%
** A40 (d)**	$-(1/k)\;ln((1-{\left(40/A\right)}^{\frac{1}{3}})/b)\;$	383.9
**Males**		
** AIP (d)**	$ln(3b)/(k+k_{\mathrm{sex}})$	46.69
** WIP (kg)**	$8(A+A_{\mathrm{sex}})/27$	14.76
** A50M (d)**	$-\ln((0.5^{\frac{1}{3}}-1)/-b)/(k+k_{\mathrm{sex}})$	97.89
** PM150d (%)**	${\left(1-be^{\left(-(k+k_{\mathrm{sex}})(150)\right)}\right)}^{3}$	67%
** A40 (d)**	$-(1/(k+k_{\mathrm{sex}}))\;ln((1-{\left(40/(A+A_{\mathrm{sex}})\right)}^{\frac{1}{3}})/b)$	213.6

AIP (d): age at the inflection point (days); WIP (kg): body weight at the inflection point (kg); A50M (d): age at 50% of maturity (days); PM150d (%): percentage of maturity at 150 days of age; A40 (d): Age at 40 kg of live weight.

## Discussion

The superior performance of the Von Bertalanffy model observed in this study aligns with previous reports indicating its suitability for describing growth patterns in sheep and other livestock species (McManus et al. 2003; Lupi et al. 2015; Mohammadi et al. 2019). The lower AIC value suggests that this model captures the biological growth trajectory of Columbia sheep more accurately than the Gompertz, Logistic, and Brody functions. García-Muñiz et al. (2019) modeled the growth curve of Boer goats; they found similar results: the model including the Von Bertalanffy function achieved better goodness-of-fit than those with the Gompertz, Brody, and Logistic functions.

The estimated asymptotic body weight indicates a moderate mature size for Columbia sheep under central Mexican production conditions. The significant sex effect observed for parameter A confirms that males achieve heavier mature weights than females, a pattern consistently reported across sheep populations (McManus et al. 2003; Lupi et al. 2015; Mohammadi et al. 2019). This difference reflects sexual dimorphism, driven by hormonal and physiological factors that influence muscle and skeletal development.

Animals with higher values of the maturity rate parameter (k) reach their mature body weight more rapidly (Sarmento et al. 2006; Lupi et al. 2016). In the present study, females exhibited a slightly higher k value than males, indicating earlier maturation. Although males matured more slowly, they ultimately achieved higher mature weights, suggesting a prolonged growth period. Similar sex-related differences in growth dynamics have been reported in other sheep breeds (Gbangboche et al. 2011; Lupi et al. 2015).

The nonlinear mixed model accommodates heterogeneity among individuals arising from variables that are not measured by including random effects (Júnior et al. 2023). The variance of random effects ($A_{i}$ and $k_{i}$) provides estimates of the growth parameter variances across Columbia sheep. Larger random effects indicate greater individual variation in growth trajectories that is not explained by the fixed effects included in the model (Boedeker 2021). The negative correlation between these parameters supports the biological trade-off between growth rate and mature size, as previously emphasized by Brown et al. (1976) and McManus et al. (2003). Animals that develop earlier tend to reach a lower adult weight, whereas those with slower maturation achieve a greater mature size.

Regarding management implications, females reached lower ages and weights at the inflection point, suggesting earlier growth deceleration than males. These results may help define age or weight targets for further evaluation of slaughter strategies, as proposed by Lupi et al. (2015). In Mexico, lambs are commonly harvested at approximately 40 kg (Hernández-Cruz et al. 2009). According to the Von Bertalanffy model, male Columbia lambs were predicted to reach 40 kg of body weight at 212 days of age (95% CI: 192–241 days), providing practical information for production planning.

## Conclusions

All four nonlinear mixed models adequately described the growth pattern of Columbia sheep. However, the Von Bertalanffy model provided the best fit for both males and females, as indicated by its lower AIC and RMSE values compared with the other models. The selected model revealed sex-related differences in growth dynamics, with males achieving greater mature body weights but at a slightly slower maturation rate than females. In addition, the derived growth indicators, including age and weight at the inflection point, percentage of maturity at 150 days of age, and the predicted age to reach 40 kg of body weight, provide biologically meaningful information that may support feeding management and decisions regarding market age under production systems similar to those evaluated in this study.

## Supplementary Material

txag100_Supplementary_Data

## Data Availability

The data that support the findings of this study are available from the corresponding author upon reasonable request.

## Abbreviations

A: Asymptotic body weight
b: Integration constant
k: Maturity rate
AIC: Akaike Information Criterion
AIP: Age at the inflection point
WIP: Body weight at the inflection point
A50M: Age at 50% of maturity
PM150d: Percentage of maturity at 150 days of age
Cor(A, k): Correlation between random effects of A and k
