# Supplementary material for: Nonlinear mixed models to describe the growth of Columbia sheep in Mexico
Source: Transl Anim Sci. 2026 Jul 17;10:txag100. doi: 10.1093/tas/txag100 (PMC13426000; doi:10.1093/tas/txag100)
Supplement: txag100_Supplementary_Data [file txag100_supplementary_data.docx]

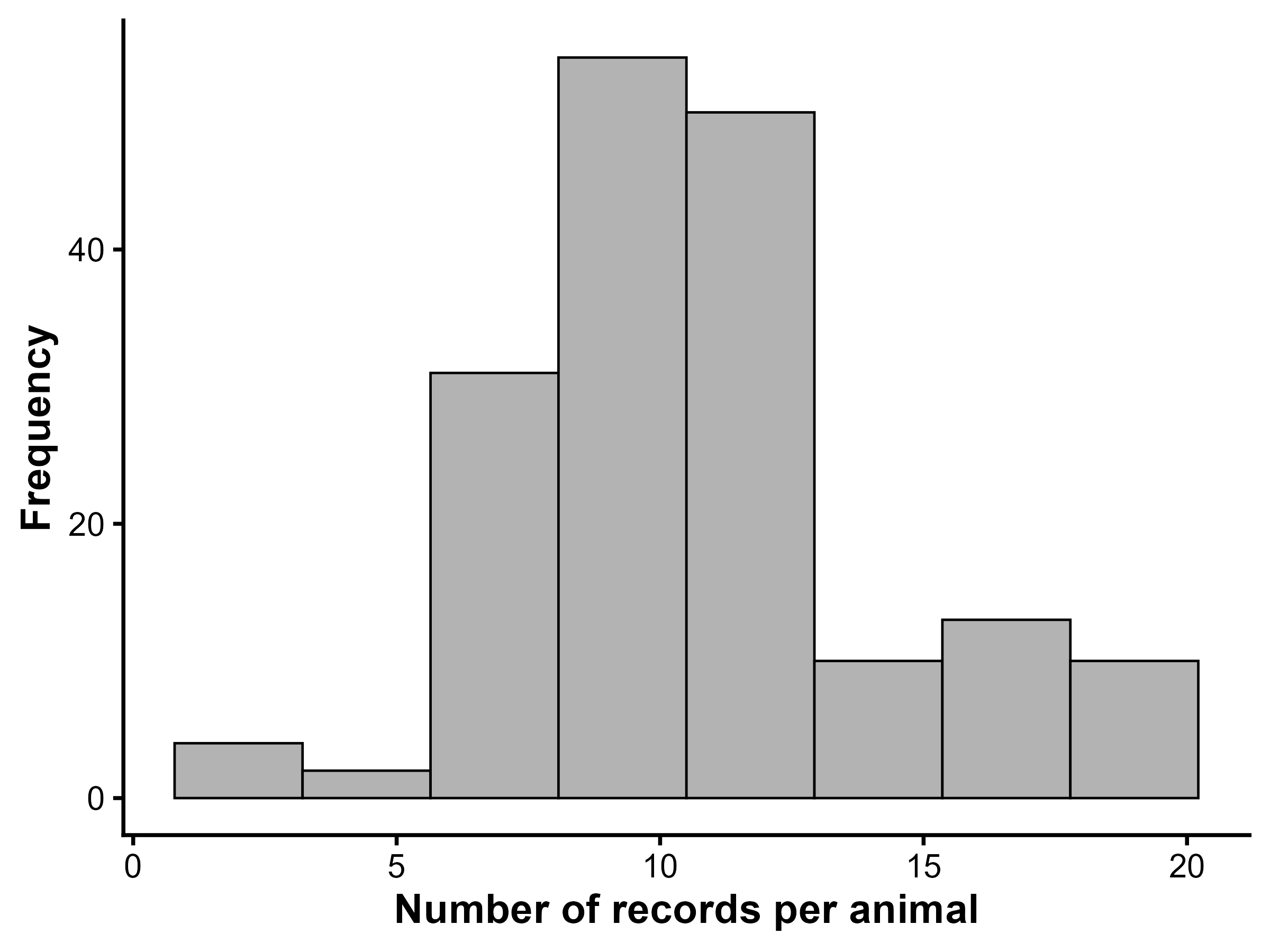


Figure S1. Distribution of the number of records per animal.


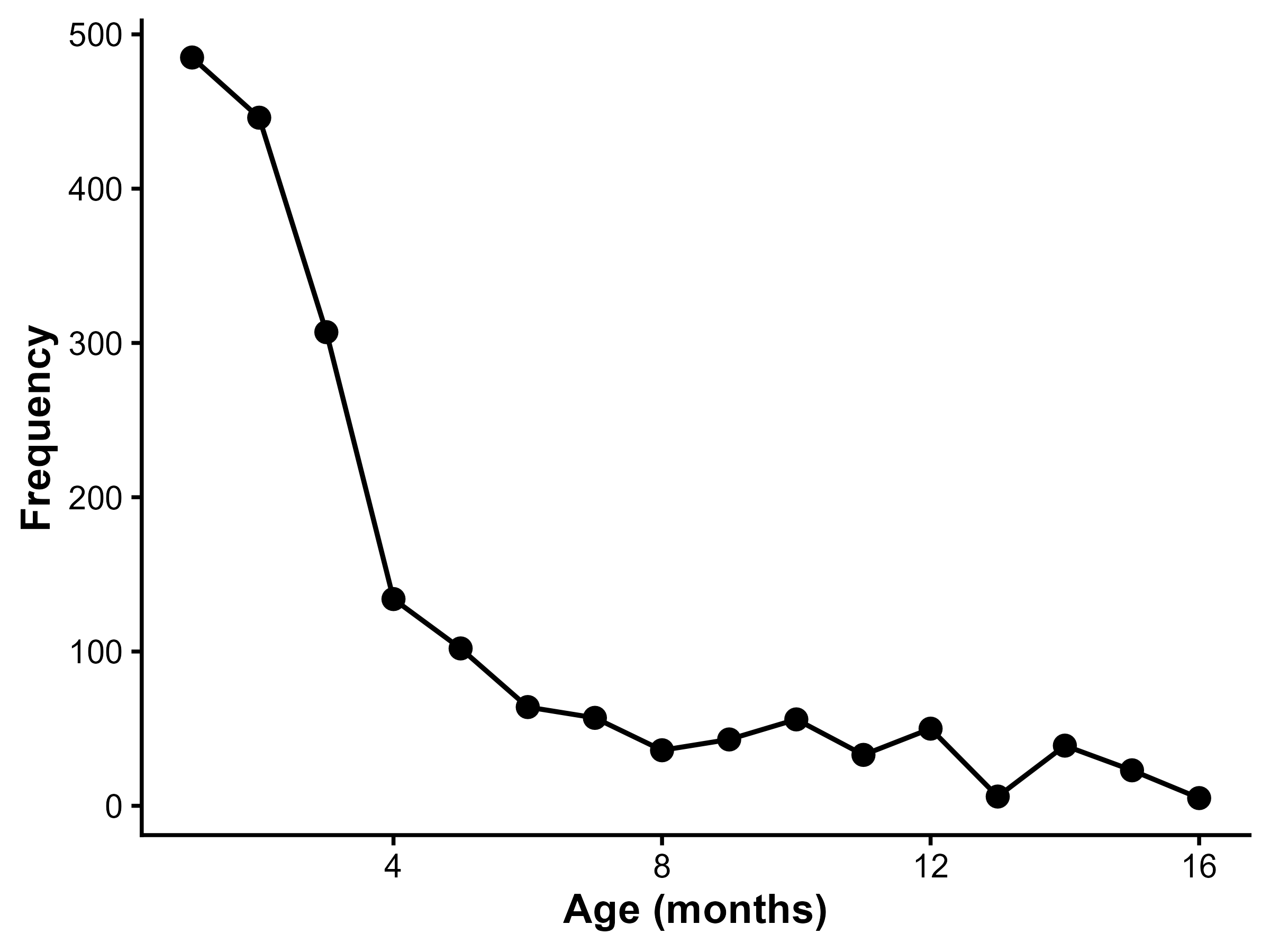


Figure S1. Distribution of sheep records by age (months).
